# Executive dysfunctions mediate between altered sensory processing and daily activity performance in older adults

**DOI:** 10.1186/s12877-021-02032-0

**Published:** 2021-02-22

**Authors:** Batya Engel-Yeger, Sara Rosenblum

**Affiliations:** Department of Occupational Therapy, Faculty of Social Welfare and Health Sciences, University of Haifa, Aba Hushi, 199, Mount Carmel, 3498838 Haifa, Israel

**Keywords:** Older adults, Executive functions, Sensory processing, Activities of daily living

## Abstract

**Background:**

Sensory processing is essential for the interaction with the environment and for adequate daily function. Sensory processing may deteriorate with aging and restrict daily activity performance. Aging may also affect Executive functions (EFs) which are critical for daily activity performance. Yet, most studies refer separately to the impacts of sensory processing or EFs and use clinical evaluations that do not necessarily reflect functional restrictions in real life. This study aims to describe the prevalence of altered sensory processing in the elderly as expressed in daily life scenarios and explore whether EFs mediate between altered sensory processing and daily activity performance in older adults.

**Methods:**

This cross-sectional study included 167 healthy independently functioning people aged 65 and above who were living in the community, had sufficient cognitive status and no symptoms of depression (based on the GDS and the MMSE). All participants completed a socio-demographic-health questionnaire, the Adolescent/Adult Sensory Profile, the Behavior Rating Inventory of Executive Function–Adult Version and the Daily Living Questionnaire.

**Results:**

Altered sensory processing, and mainly by the reduced ability to register and modulate sensory input from daily environment, were prevalent in older adults. Their impacts on daily activity performance were mediated by executive dysfunctions.

**Conclusions:**

Executive dysfunctions may worsen the negative effects of altered sensory processing on daily activity performance in older adults. The interaction between EFs and sensory processing should receive growing attention in intervention and prevention programs for older adults, with the emphasis on their expressions and implications on peoples’ function in real life context.

## Background

Sensory processing refers to the way individuals manage incoming sensory information, including its reception, modulation and integration with other brain areas and networks, to produce an adaptive output to environmental demands [1]. Since sensory systems provide the brain with ongoing information about the environment, adequate sensory processing is fundamental for proper cognitive and emotional function, for accurate movements, for learning, for socializing and for other activities that characterize our daily lives [2].

Altered sensory processing refers to difficulties to modulate and organize the intensity of response to sensory input in a graded and adaptive manner. Altered sensory modulation result in hypo or hypersensitivity to sensory input. Individuals with hypersensitivity respond to sensation faster, intensely and for a longer duration. They are overwhelmed with sensation and even experience “regular” stimuli as painful. This may lead to behavioral dysregulation expressed in stressful behavior, anxiety, irritability and avoidance from interactions with the physical and the social environments [1]. On the contrary, individuals with hyposensitivity tend to miss sensory stimuli and thus fail to respond accurately to environmental demands [1]. Both hyper or hypo sensitivity may restrict adaptive behavior [2] and interfere with daily activity performance [3].

One of the theoretical models that refers to the interaction between the neurological-threshold to sensory input and the related behavioral regulation is Dunn’s model [4]. According to this model, the interaction between the neurological threshold and behavioral regulation results in four patterns: (1) Low registration - people with high neurological thresholds (hyposensitive) and passive behavioral strategies that fail to detect sensory information and do not seek for it; (2) Sensory seekers - people with high neurological threshold who actively seek for rich and intense sensory stimuli (3) Sensory sensitivity – people with low neurological thresholds (hypersensitive) and passive behavioral, meaning they do not actively eliminate uncomfortable sensations; thus, they frequently experience discomfort, distraction, and emotional burden (4) Sensory avoiding - people with low neurological thresholds who actively limit their exposure to unpleasant sensations.

The very few studies that used Dunn’s model in older people, found that older people tend to show lower registration of sensory input, lower tendency to seek for sensory input and in some cases have modulation difficulties that result in hyper sensitivity. These alterations were found to significantly interfere with daily activity performance, with participation in daily situations and reduce quality of life [5, 6].

Nevertheless, most studies about sensory processing and modulation in the elderly focus on sensory acuity or sensory impairment in a specific sensory modality, mainly visual, auditory and vestibular [7–11], as measured in laboratory settings. Less is known about the ability of elderly people to process and modulate sensory information from various modalities, in daily life scenarios [5]. Moreover, the literature emphasizes that the aging brain tends to rely increasingly on multisensory integration rather than on a unisensory modality to compensate for the neural and sensory deterioration [12]. Hence, studies should further explore alterations in sensory processing and modulation in older adults, as expressed in all modalities and in daily life scenarios, in order to create better intervention programs, that are not relevant only for the clinical setting, but may be applicable to daily environments. For that, studies should also elucidate the contribution of relevant factors responsible for behavioral regulation that affect the adaptive responses to environmental demands, such as the high cognitive abilities named executive functions (EFs) [13–15].

The high cognitive abilities responsible for behavioral regulation, for formulating goals, planning how to achieve them, and carrying them out effectively [16] named EFs, are extremely affected by aging and significantly interfere with daily activity performance [17]. Decline in EFs in older adults, as a result from neurodegenerative changes [18] is related to deficient performance of instrumental activities of daily living (IADL), and impairment in elementary activities that enable keeping daily routines, such as: driving, money management, shopping [19–21]. The evaluation of EFs in the elderly is also challenging – in most cases EFs are measured by laboratory neuropsychological/cognitive measures, which mainly focus on specific aspects of EFs such as inhibition; shifting, etc. with no referral to the implications on people’s daily function [22, 23]. In order to better reflect the implications of EFs on daily activities, ecological valid assessments that imitate daily scenarios are needed [24–26]. One of the efficient ecological valid assessment which evaluates the multicomponent expressions of the EFs in daily life scenarios is the Behavior Rating Inventory of Executive Function–Adult version (BRIEF-A) [27].

Yet, very few studies refer to the association between sensory processing and EFs. The existing data collected on populations with central nervous system abnormalities such ADHD, brain injuries and stroke, found that altered sensory processing was related to reduced EFs expressed for example in attentional problems, impaired working memory and inhibition problems [28–31].

Although studies show that altered sensory processing in the elderly affects daily activity performance, and may be related to executive dysfunctions, less is known about the contribution of EFs to the impacts of altered sensory processing on daily activities. Understanding how the ability to regulate behavior, manage actions and decisions modulates the connection between altered sensory processing and daily activity performance is critical for intervention programs. By understanding the role of sensory processing, EFs and the relationships between them, in determining functional resilience or vulnerability, prevention and intervention programs may improve daily function and quality of life in the elderly. Based on the above, this study aimed to profile altered sensory processing in older adults, in all sensory modalities, as expressed in their daily life and to explore whether EFs mediate the relationship between sensory processing and the individual’s ability to perform daily activities. It was hypothesized that EFs indeed mediate the relationship between sensory processing and daily activity performance.

## Methods

### Participants

According to Power analysis, with effect size of .20, *p* = .05, and power of .95, a sample size of 131 participants was recommended. Our sample included 167 healthy and independently functioning people 65 years and older who lived in the community. All participants for this cross-sectional study were recruited via advertisements published in community centers and via contacts with managers of senior-citizen homes. All participants had functional independence and lived in their private or senior-citizen homes. They were proficient in their native language (speaking, reading, and writing), were right-handed, had normal vision and hearing ability (with or without correction) and had at least 8 years of education (because of life circumstances related to the country, part of the elderly population could not complete high school. However, all study participants with 8 to 12 years of education had well independent daily functioning).

Exclusion criteria included presence of systemic severe chronic diseases such as cancer; severe nervous system impairments, such as cerebrovascular accident, Parkinson’s disease, or untreated diabetes; and depression. An initial screening using the Mini-Mental State Examination (MMSE) [32] revealed that all the participants had sufficient cognitive status based on their score of 24 or above [33] and no symptoms of depression according to the Geriatric Depression Scale [34, 35] (GDS). Table 1 summarizes participants’ sociodemographic information.

**Table 1:** Participants’ Sociodemographic Information (N = 167)

Variable	%		
Gender			
Man	44.3		
Woman	55.7		
Socio-economic level			
Low	12.0		
Average	68.3		
High	14.4		
Missing data	5.3		
Type of living			
Private home	86.8		
Senior-citizen home	13.2		
	*M*	*SD*	Range
Age (years)	74.24	8.05	65–100
Education (years)	14.73	2.71	8–25
Mini Mental State Examination score	28.74	1.16	26–30

### Measures

In addition to initial participants’ screenings with the MMSE [32] and the GDS [34, 35], participants completed a socio-demographic-health-status questionnaire, and the following measurements:

#### The adolescent/adult sensory profile (AASP) [36]

This self-report tool is based on Dunn’s model [4] and includes 60 questions about the respondent’s behavior with sensory experiences in daily living in all sensory modalities. The 60 items are sorted equally into the four quadrants of Dunn’s model—low registration, sensation seeking, sensory sensitivity, and sensation avoiding (based on factor analysis)—and reflect various sensory-processing patterns. Each quadrant provides information on multiple sensory systems. Participants indicate how often they respond to the sensory event in the manner described using a 5-point Likert scale from 1 (almost never) to 5 (almost always). Each quadrant’s resultant score can range from 5 to 75. Norms are described for each age group (11–17 years; 18–64; 65 and older). Scores under or above these norms are considered alterations in sensory-processing abilities. This questionnaire has good internal consistency with coefficient alpha values of .692 to .699 for the four quadrants. In the present study the validated Hebrew version was used [37].

#### The behavior rating inventory of executive function-adult version (BRIEF-A) [27]

This is a standardized rating scale developed to assess everyday behaviors associated with specific EF domains in adults (18–90 years). It consists of equivalent self-report and informant-report forms, each having 75 items in nine non-overlapping scales; two summary index scales—the behavioral regulation index (BRI) and the metacognition index (MI); and a global executive composite (GEC) scale that reflects overall functioning. The BRI is composed of four scales: Inhibit, Shift, Emotional Control, and Self-Monitor. The MI is composed of five scales: Initiate, Working Memory, Plan/Organize, Task Monitor, and Organization of Materials. The BRIEF-A can serve as a screening tool for possible executive dysfunction and as an indicator of individuals’ awareness of their own self-regulatory functioning. Behavior frequency is rated on a Likert scale from rare to often. Raw scores are transferred into t scores (M = 50, SD = 10). T scores at or above 65 are considered clinically significant. In this study, we used the self-report form. The BRIEF has a validated Hebrew version which was used in this study [28].

#### The daily living questionnaire (DLQ) [38]

This is a 56-item self-report developed to evaluate IADL performance with a focus on their relation to cognitive abilities. The respondents report how much mental difficulty they have when performing varied tasks. Part 1 addresses activities and participation (e.g., household tasks, community/participation, and complex tasks). Part 2 addresses cognitive symptoms or impairments (e.g. in working memory, multitasking, and organization). Example items include “Getting ready in the morning” and “Finding items on a crowded shelf or closet.” Respondents score each item as 1 (*no difficulty*), 2 (*some difficulty*), 3 (*much difficulty*), 4 (*unable to do*), or not applicable (item is not rated). Four additional questions focus on the participant’s perception of their general functional and cognitive abilities from 1 (*very good*) to 5 (*weak*). Participants also rate their degree of satisfaction with their ability to perform what they need and want to do in everyday life from 1 (*satisfied*) to 5 (*dissatisfied*). High internal consistency has been indicated for the two parts (.88–.92). In this study we used the Hebrew version of the DLQ [38].

### Procedure

The study was performed based on the ethics approval of the Institutional Review Board of the Israeli Ministry of Health (Study number: 3–5861) and after participants singed the consent to participate in this study. Those who answered the advertisements calling to participate in this study, contacted the research coordinators by phone. In this conversation participants received an elaborated information about the study and a home meeting was set with them. In this meeting participants signed the consent form and completed the socio-demographic-health questionnaire, the GDS, the MMSE, the AASP, the BRIEF-A and the DLQ.

### Statistical analysis

All analyses were performed using the Statistical Package for Social Sciences (SPSS) for Windows 25.0. Descriptive statistics was conducted for all measures in the sample. Chi-square test examined between-group differences in prevalence distribution for the relevant variables. Multivariate analysis of variance examined whether significant between-group differences existed in all dependent measure subscales. Further analyses of variance evaluated the significance of the between-group differences in the total scores of all dependent measures. Bonferroni correction was included.

Structural equation modeling (SEM) [39] was used to examine relationships among age, sensory profiles (AASP), executive functions (BRIEF-A), and cognitive deficits in IADL performance (DLQ). In addition, goodness-of-fit statistic, goodness-of-fit index, root-mean-square error of approximation (RMSEA), standardized root-mean-square residual, and comparative fit index (CFI) were tested. Here, Chi-square was used for nested model comparisons. The level of significance was set at .05.

## Results

To understand the participants’ characteristics better, we first describe their sensory processing abilities, EF, and cognitive deficits in daily activities, as measured by the AASP, BRIEF, and DLQ, respectively.

### Sensory processing

As presented in Table 2, most participants had normal sensory-processing abilities and were found to be in the normal range of each sensory profile of the AASP.

**Table 2:** Sensory Processing Patterns: Descriptive Statistics and Prevalence

Sensory processing pattern	Minimum	Maximum	*M*	*SD*	Normal range	%
Low registration	15	49	28.91	6.842	27–40	
Under norm						24.0
Norm						51.5
Above norm						9.0
Missing						15.6
Seeking	22	69	45.26	7.660	40–52	
Under norm						24.6
Norm						52.7
Above norm						8.4
Missing						14.4
Sensitivity	15	56	33.43	8.520	26–41	
Under norm						17.5
Norm						65.7
Above norm						16.8
Avoiding	18	57	33.93	8.510	26–42	
Under norm						17.4
Norm						58.1
Above norm						10.2
Missing						14.4

### Executive dysfunctions

Table 3 depicts the sample’s EF as measured by the BRIEF-A. The mean scores describe normal values. When measuring the prevalence of executive dysfunctions, most (77%) participants had normal EF (i.e., scored less than 65), whereas 23% had behavioral dysregulation/executive dysfunctions (scored 65 or more).

**Table 3:** Participants’ Behavioral Regulation/Meta-Cognition (Executive Functions) (BRIEF-A T-Scores)

BRIEF-A subscale	Minimum	Maximum	*Mean*	*SD*
Inhibition	40	82	51.29	8.07
Shift	40	79	54.33	9.54
Emotional control	40	95	59.39	12.42
Self-monitor	38	95	51.43	11.21
Initiate	38	77	50.62	9.52
Working memory	40	88	53.67	11.51
Plan/organize	40	86	52.12	11.07
Task monitor	38	98	53.18	10.58
Organization of materials	38	93	49.38	11.18
BRI	36	100	56.01	11.14
MI	37	81	52.09	10.58
GEC	37	88	55.95	10.35

*Note. BRI* Behavioral Regulation Index; *MI* Meta-Cognition Index; *GEC* Global Executive Composite

*SD* standard deviation

### Daily activities performance

Table 4 depicts participants’ cognitive abilities expressed in IADL performance as measured by the DLQ. Scores ranged from 1 (*no difficulty*) to 3 (*much difficulty*).

**Table 4:** Participants’ Cognitive Abilities Expressed in IADL (DLQ)

DLQ factor	Minimum	Maximum	*Mean*	*SD*
Household	1.00	2.43	1.21	.29
Language	1.00	3.00	1.23	.33
Community participation	1.00	2.43	1.28	.36
Complex tasks	1.00	2.71	1.41	.43
Multitask organization	1.00	2.73	1.47	.44
Memory	1.00	3.00	1.26	.36
Monitor	1.00	2.22	1.29	.33
Activities participation	1.00	2.29	1.28	.29
Cognitive symptoms	1.00	2.42	1.36	.34

*Note.* 1 = no difficulty, 2 = some difficulty, 3 = much difficulty

*SD* standard deviation

### Differences in EF between people with and without altered sensory processing

According to Aim 1, we examined differences in EF (BRIEF-A) and cognitive deficits in IADL performance (DLQ) between participants with and without altered sensory processing. Those with altered sensory processing showed reduced EF, as measured by most BRIEF scales, than did those in normal ranges of the AASP quadrants. As shown in the mean differences, people with lower ability to register sensory input scored significantly lower in inhibition (*M* = 7.69, *p* = .001), shifting efficiency (*M* = 8.18, *p* = .004), working memory (*M* = 9.31, *p* = .007), behavioral regulation (*M* = 9.93, *p* = .001), and GEC (*M* = 7.53, *p* = .01) than did those with normal registration.

No significant differences in BRIEF-A scores were found between people in the sensory-seeking profile ranges. However, as described here by mean differences, participants with extreme sensitivity to sensory input had significantly lower scores in inhibition (*M* = 6.78, *p* < .0001), initiation (*M* = 6.68, *p* = .003), working memory (*M* = 9.77, *p* < .0001), plan/organize (*M* = 9.46, *p* < .0001), task monitor (*M* = 6.02, *p* = .03), behavioral regulation (*M* = 7.35, *p* < .0001), meta-cognition (*M* = 8.69, *p* < .0001), and GEC (*M* = 8.19, *p* < .0001) than did those in the normal range of sensory sensitivity.

People with extreme sensory avoidance scored significantly lower, as shown by mean differences, in inhibition (*M* = 7.64, *p* = .001), emotional control (*M* = 8.50, *p* = .02), initiation (*M* = 9.11, *p* < .0001), working memory (*M* = 12.05, *p* < .0001), plan/organize (*M* = 14.37, *p* < .0001), task monitor (*M* = 7.51, *p* = .02), behavioral regulation (*M* = 8.55, *p* = .005), meta-cognition (*M* = 10.37, *p* < .0001), and GEC (*M* = 10.11, *p* < .0001) than did those in the normal range of sensation avoidance.

**Fig. 1:**
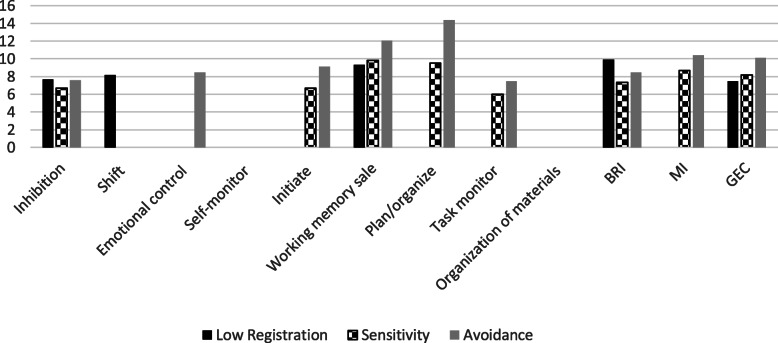
Mean Differences between Participants with Extreme and Normal Sensory Processing (BRIEF-A). Note. BRI = Behavioral Regulation Index; MI = Metacognition Index; GEC = Global Executive Composite

Figure 1 depicts the visual presentations of the mean scores of each BRIEF-A scale in each sensory processing pattern.

### Differences in daily activities performance between people with and without altered sensory processing

Participants with altered sensory processing showed greater deficits in IADL performance (as measured by the DLQ) than did those scoring in the normal ranges of the AASP quadrants:

participants with lower ability to register sensory input had more difficulties to perform activities related to household (mean difference = .24, *p* = .006). Participants with greater sensory sensitivity had more difficulties to perform activities as reflected by the following DLQ scales: language (*M* = .33, *p* < .0001), community/participation (*M* = .22, *p* = .02), complex tasks (*M* = .28, *p* = .01), multitask organization (*M* = .34, *p* = .002), memory (*M* = .31, *p* < .0001), monitoring (*M* = .35, *p* < .0001), activity participation (*M* = .23, *p* = 001), and cognitive symptoms (*M* = .33, *p* < .0001) compared to those in those found in the normal sensory sensitivity range.

**Fig. 2:**
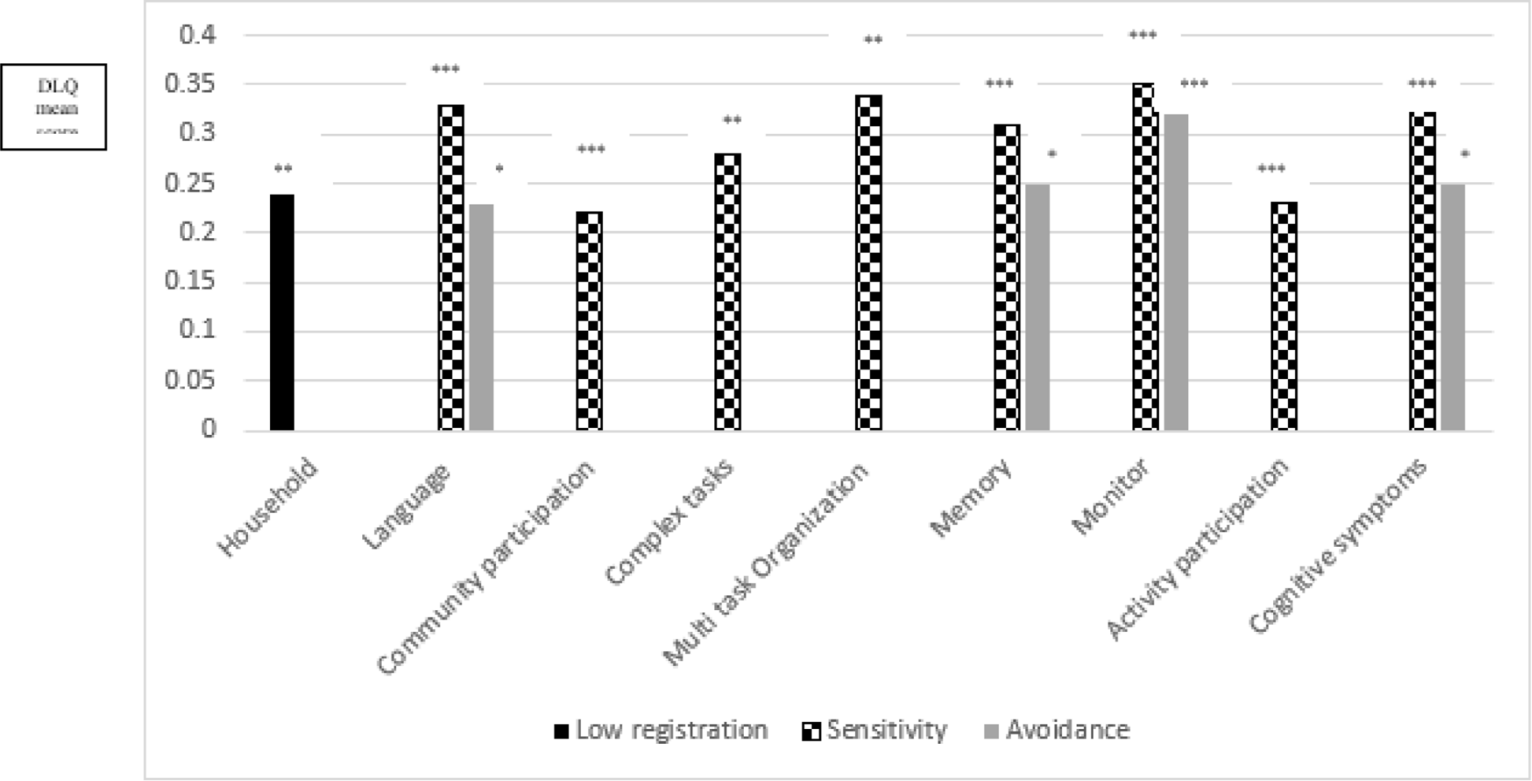
Mean Differences between Participants with Extreme and Normal Sensory Processing in each DLQ section. Note. *p ≤ .050; **p ≤ .010; ***p ≤ .00

Participants with extreme sensation avoidance also had greater to perform activities as reflected by the following DLQ scales: language (*M* = .23, *p* = .04), memory (*M* = .25, *p* = .02), monitor (*M* = .32, *p* < .0001), and had greater cognitive symptoms (*M* = .25, *p* = .02) than those found in the normal sensory avoidance range (see Fig. 2).

### Relationships between age, EF, sensory processing and daily activities performance

Based on the literature that emphasizes the relationships between aging, worse sensory processing, executive dysfunction and restricted ability to perform daily activities, together with the results described above, a SEM model was created. This SEM model aimed to provide detailed information about the relations between age, the four sensory processing patterns presented in Dunn’s model, the EF as measured by the BRIEF, and examine whether EF mediate between sensory processing and daily activity performance.

The SEM model revealed good fit indices, including normed fit index (NFI), χ^2^(15) = 21.68, *p* = .12; CFI = .98; NFI = .97; RMSEA = .05. There was a direct effect between age and sensory seeking (β = −.20, *p* = .009), in which older participants had less tendency to seek sensory input in their daily environment. The effect between age and low registration was close to significant (β = .13, *p* = .08), as was the effect between age and sensory avoiding (β = .14, *p* = .06). Hence, these results show a tendency for the older participants to have higher avoidance and greater difficulty registering sensory input. Nevertheless, low registration and sensory sensitivity were directly related to BRIEF-A scores (β = .32, *p* < .001; β = .40, *p* < .001 respectively). This means that greater difficulties with register sensory input correlated with worse EF (BRIEF-A) and with greater cognitive deficits expressed in IADL performance (DLQ). Further, the BRIEF-A was directly related to the DLQ total score (β = .39, *p* < .001), but no significant indirect effect was found between age and BRIEF-A scores or between age and DLQ scores.

Sensory sensitivity and low registration had significant indirect effect to the DLQ (.16 and .12, respectively) mediated by the BRIEF-A for sensitivity (*p* = .001) and low registration (*p* = .002), 95% CIs [.07, .31], [.04, .27], respectively (see Fig. 2). This model explains 46% of the BRIEF-A variance and 42% of the DLQ variance.

**Fig. 3:**
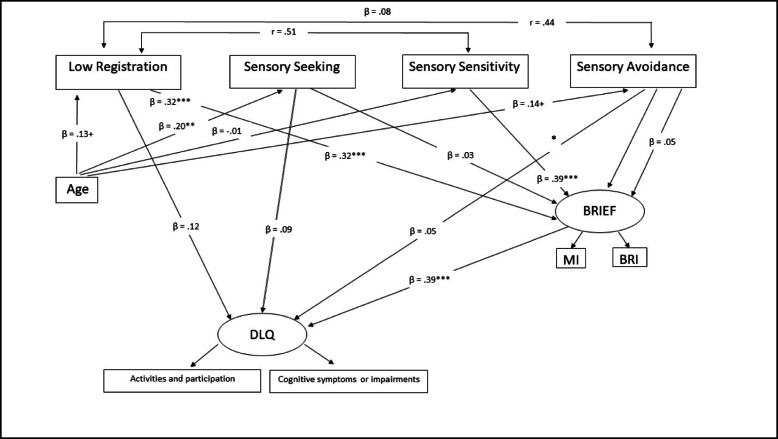
Structural Equation Modeling: Relationship between Age, Sensory Processing, Executive Functions, and Daily Activity Performance. Note. *N* = 167, χ^2^(15) = 21.68, *p* = .12, CFI = .98, NFI = .96, RMSEA = .05. ^+^*p* < .100; ^*^p ≤ .050; ^**^p ≤ .010; ^***^p ≤ .001

In sum, older people had less tendency to seek sensory input in their daily environment. Their greater difficulties registering sensory input correlated with worse EF (BRIEF-A) and greater cognitive deficits expressed in IADL (DLQ). Sensory sensitivity and low registration had significant indirect effects with DLQ mediated by BRIEF-A (Fig. 3). Hence, executive dysfunctions may worsen the negative effects of altered sensory processing on daily activity performance in older adults.

## Discussion

This study focused on altered sensory processing, executive dysfunctions and their impacts on daily activity performance in older adults. The main findings are that (a) older adults have less efficient sensory processing and reduced EFs, as expressed in their daily life scenarios (b) EFs mediate the relation between altered sensory processing and daily activity performance. This means that, even if sensory processing is altered in older adults, it is the better or worse EFs that define their level of impact on daily activity performance.

When referring to the specific sensory processing profiles - most participants scored within the normal range of each AASP sensory profile. Since this study’s sample included relatively healthy and functional older adults, the sample appears representative of sensory processing in the older population. However, when examining the prevalence of participants with extreme sensory processing, according to the AASP, 9% had difficulties registering sensory input and about 25% had lower tendency to seek for sensory input. Indeed, older adults may require a greater amount of sensory input to react. If this amount is not provided, it may lead to lower awareness of the environment [39, 40, 41]. A similar prevalence of above- and below-AASP norms was noted in sensory patterns with low neurological thresholds (sensory sensitivity and avoidance), highlighting the modulation difficulties.

Whether sensory input from the daily environment is experienced as overwhelming, or, on the contrary, is missed because of hyposensitivity, other abilities - physical, cognitive, and emotional - may decline [42–44]. Thus, the individual’s interaction with the environment may be impaired and restrict the engagement in daily occupations and activities. These functional limitations may contribute to social withdrawal [1] and isolation [45] known to be prevalent in the elderly and severely reduce quality of life and well-being [46]. Thus, prevention programs should screen for sensory-processing alterations in older adults and understand their implications on daily life and on the persons’ well-being.

The second significant outcome of this study emphasizes that referring only to sensory processing is insufficient. Science and practice should also consider other key factors that may affect the interactions between the individual and his/her physical and social environments. In this study, the assumption was that hyper or hypo sensitivity to sensory input does not stand alone as a factor that influences the interaction with the environments and the performance of daily activities. As mentioned in Dunn’s model [4], it is the interaction with the person’s ability to regulate his/her behavioral response to the sensory input, that contributes to activity performance. Indeed, in this study, the high cognitive abilities named EFs, that are responsible for an individual’s-controlled goal-oriented behavior mediated between sensory processing and daily activity performance. Executive dysfunctions, as expressed in daily life situations, were found in about one-third of the sample. This result highlights several points: first, the vulnerability for executive dysfunctions warrants attention also among “normal” older adults. Second, due to the EFs domain’s complexity, it is not enough to evaluate separate EFs components in the lab. A transition to real- life context is necessary. For that, appropriate performance-based measures should be used to evaluate EFs expressions and functional impacts in the real world [47]. Evaluations such as the BRIEF-A and DLQ provide clinicians with practical functional knowledge that bridges clinical observations with people’s function in their natural environments. Third, the analysis of the SEM model showed that altered sensory processing was associated with reduced EFs as reflected in behavioral regulation and in meta-cognition measured by the BRIEF, supporting the study hypothesis. Neuro-imaging findings explain this association by referring to the connection between the prefrontal cortex (which plays a central role in EFs), the sensory cortical areas and the thalamic reticular subnetworks [30, 48, 49]. However, additional implications should be derived. First, the SEM model highlights the role of different facets of each of the EFs domains. For example, the interaction between AASP and BRIEF-A scores was most emphasized in sensory patterns related to passive strategies in Dunns’ model (low registration and sensory sensitivity). Moreover, low registration and sensory sensitivity had significant indirect effect on DLQ, mediated by the BRIEF-A. This may suggest that people who use passive strategies to deal with sensory input are more vulnerable and have less efficient coping mechanisms, which in turn, restrict their daily activity performance. On the other hand, individuals who seek for sensory input and enjoy it in their daily life, experience better well-being and quality of life [50]. This point should be examined in further studies.

Prevention and intervention programs for older adults should refer to both sensory processing and EFs [51]. Given that sensory-processing alterations worsen with age, earlier screening is a significant preventive aid. The relationships between altered sensory processing and EFs (meta-cognition and behavioral regulation) and their influence on daily activity performance should receive growing attention in clinical programs for older adults. For that, assessments with ecological validity that reflect how age-related dysfunction are expressed in people’s real lives and affect daily performance are most relevant. These assessments may elevate the individuals’ awareness to their dysfunctions. Individuals may better notice when and how their dysfunctions interfere with daily activity performance. With this knowledge, clinicians may suggest strategies to deal with difficulties related to altered sensory processing and EFs, and overcome obstacles in daily activity performance [52]. These strategies promise better intervention outcomes in terms of enhanced participation in daily settings and better quality of life, which are the main measures of intervention efficiency according to the World Health Organization [53].

## Conclusions

Altered sensory processing and executive dysfunctions may be prevalent in older adults. Among older adults with altered sensory processing, better or worse EFs may define the level of daily activity performance. Thus, evaluations of sensory processing should be proceeded in measuring EFs. By using measures with ecological validity, clinicians may better understand the expressions and impacts of altered sensory processing and executive dysfunctions on people’s daily life. This information may be useful for focusing intervention goals and strategies based on the individual’s specific profiles and needs in real life context.

### Limitations

This study included older adults who represent “normal aging,” meaning they are relatively healthy and functional. Future studies should explore aging impacts on sensory processing and EFs as expressed in daily lives of “normal” older populations, as well as populations with difficulties or specific health conditions. Sociodemographic effects as gender and socio-economic level should be also considered. Longitudinal studies are also recommended to examine the efficiency of prevention and intervention programs that focus on sensory and cognitive/EFs aspects in enhancing daily function, health, and well-being in the elderly.

## Data Availability

The datasets used and/or analyzed during the current study available from the corresponding author on reasonable request.

## Abbreviations

EFs: Executive functions
BRIEF-A: The Behavior Rating Inventory of Executive Function–Adult version
MMSE: The Mini-Mental State Examination
GDS: Geriatric Depression Scale
AASP: The Adolescent/Adult Sensory Profile
DLQ: The Daily Living Questionnaire
SEM: Structural equation modeling
